# Author Correction: Limited oxygen in standard cell culture alters metabolism and function of differentiated cells

**DOI:** 10.1038/s44318-024-00230-1

**Published:** 2024-09-06

**Authors:** Joycelyn Tan, Sam Virtue, Dougall M Norris, Olivia J Conway, Ming Yang, Guillaume Bidault, Christopher Gribben, Fatima Lugtu, Ioannis Kamzolas, James R Krycer, Richard J Mills, Lu Liang, Conceição Pereira, Martin Dale, Amber S Shun-Shion, Harry JM Baird, James A Horscroft, Alice P Sowton, Marcella Ma, Stefania Carobbio, Evangelia Petsalaki, Andrew J Murray, David C Gershlick, James A Nathan, James E Hudson, Ludovic Vallier, Kelsey H Fisher-Wellman, Christian Frezza, Antonio Vidal-Puig, Daniel J Fazakerley

**Affiliations:** 1 Metabolic Research Laboratories, Wellcome-Medical Research Council Institute of Metabolic Science, University of Cambridge, Cambridge, CB2 0QQ UK; 2 MRC Cancer Unit, University of Cambridge, Cambridge Biomedical Campus, Cambridge, CB2 0XZ UK; 3 CECAD Research Center, Faculty of Medicine, University Hospital Cologne, Cologne, 50931 Germany; 4 Wellcome-MRC Cambridge Stem Cell Institute, University of Cambridge, Cambridge, CB2 0AW UK; 5 European Molecular Biology Laboratory, European Bioinformatics Institute, Wellcome Genome Campus, Hinxton, CB10 1SD UK; 6 QIMR Berghofer Medical Research Institute, Brisbane, Queensland 4006 Australia; 7 Faculty of Health, School of Biomedical Sciences, Queensland University of Technology, Brisbane, Queensland 4000 Australia; 8 Cambridge Institute for Medical Research, University of Cambridge, Cambridge, CB2 0XY UK; 9 Department of Physiology, Development and Neuroscience, University of Cambridge, Cambridge, CB2 3EL UK; 10 Centro de Investigacion Principe Felipe, Valencia, 46012 Spain; 11 Cambridge Institute of Therapeutic Immunology and Infectious Disease (CITIID), Jeffrey Cheah Biomedical Centre, Department of Medicine, University of Cambridge, Cambridge, CB2 0AW UK; 12 Faculty of Medicine, School of Biomedical Sciences, The University of Queensland, Brisbane, QLD 4072 Australia; 13 Department of Physiology, Brody School of Medicine, East Carolina University, Greenville, NC 27834 USA; 14 East Carolina Diabetes and Obesity Institute, East Carolina University, Greenville, NC 27834 USA; 15 UNC Lineberger Comprehensive Cancer Center, University of North Carolina at Chapel Hill School of Medicine, Chapel Hill, NC 27599 USA

## Abstract

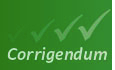

**Correction to:** *The EMBO Journal* (2024) 43: 2127–2165. 10.1038/s44318-024-00084-7 | Published online 5 April 2024

**A subheading in the Results section of the paper is corrected**.

A subheading in the Results section of the paper is corrected from:

Lowering medium volumes induces a widespread transcriptional response reminiscent of physiological hypoxia

To: (Changes in bold)

**Standard medium volumes drive** a widespread transcriptional response reminiscent of physiological hypoxia

This change does not affect the manuscript or its conclusions.

