# Supplementary figures and images for: Limited oxygen in standard cell culture alters metabolism and function of differentiated cells
Source: EMBO J. 2024 Apr 5;43(11):4. doi: 10.1038/s44318-024-00084-7 (PMC11148168; doi:10.1038/s44318-024-00084-7)

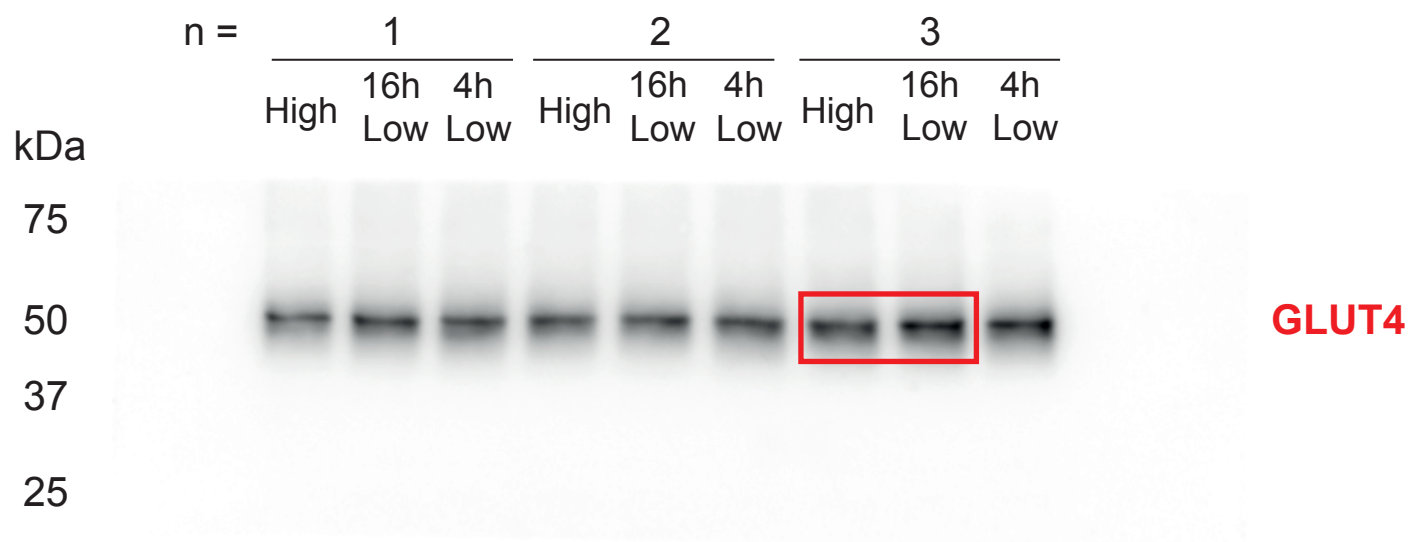

Supplement: Supplementary file 8 — Source data Fig. 2 [file 44318_2024_84_MOESM8_ESM.zip › Figure 2/2A/Western GLUT4.pdf]

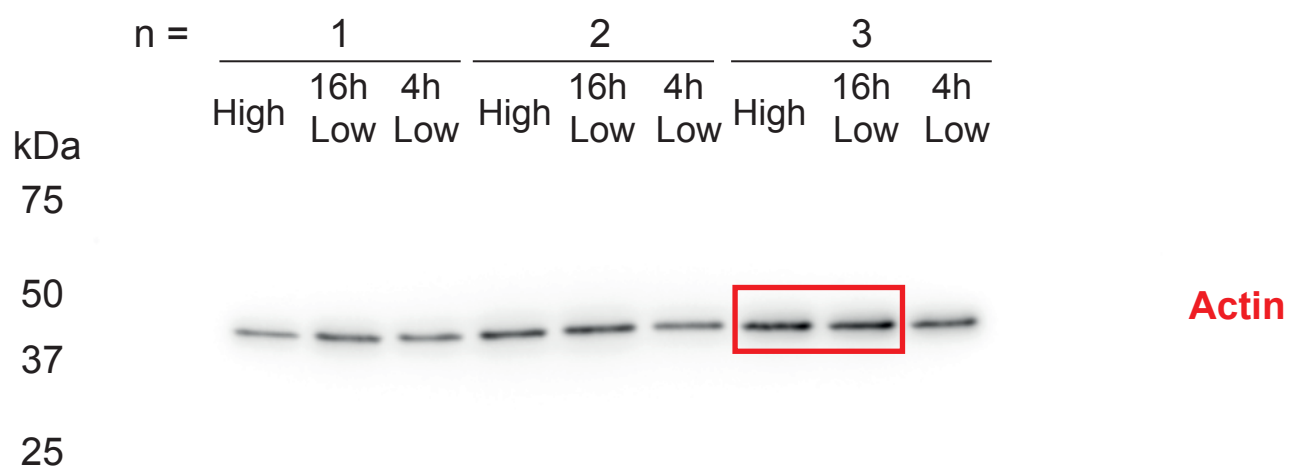

Supplement: Supplementary file 8 — Source data Fig. 2 [file 44318_2024_84_MOESM8_ESM.zip › Figure 2/2A/Western Actin.pdf]

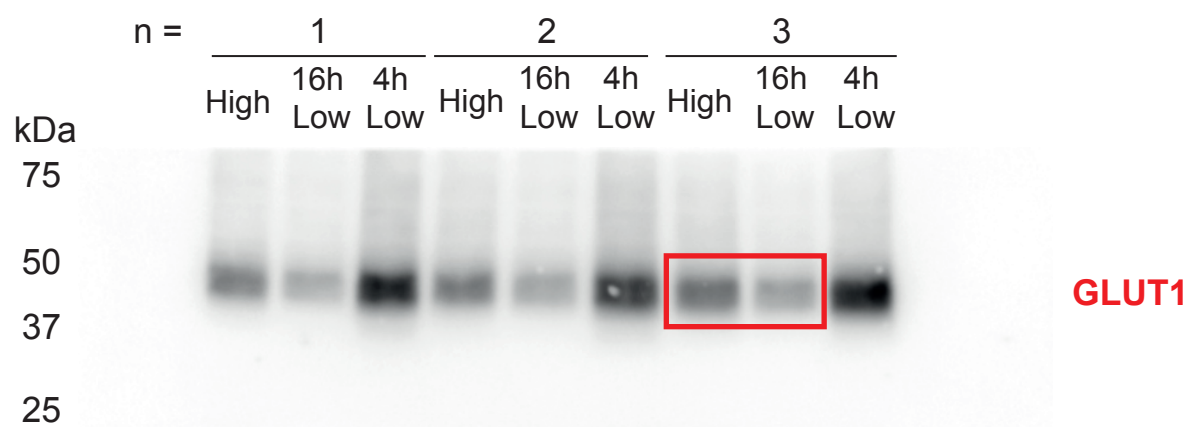

Supplement: Supplementary file 8 — Source data Fig. 2 [file 44318_2024_84_MOESM8_ESM.zip › Figure 2/2A/Western GLUT1.pdf]

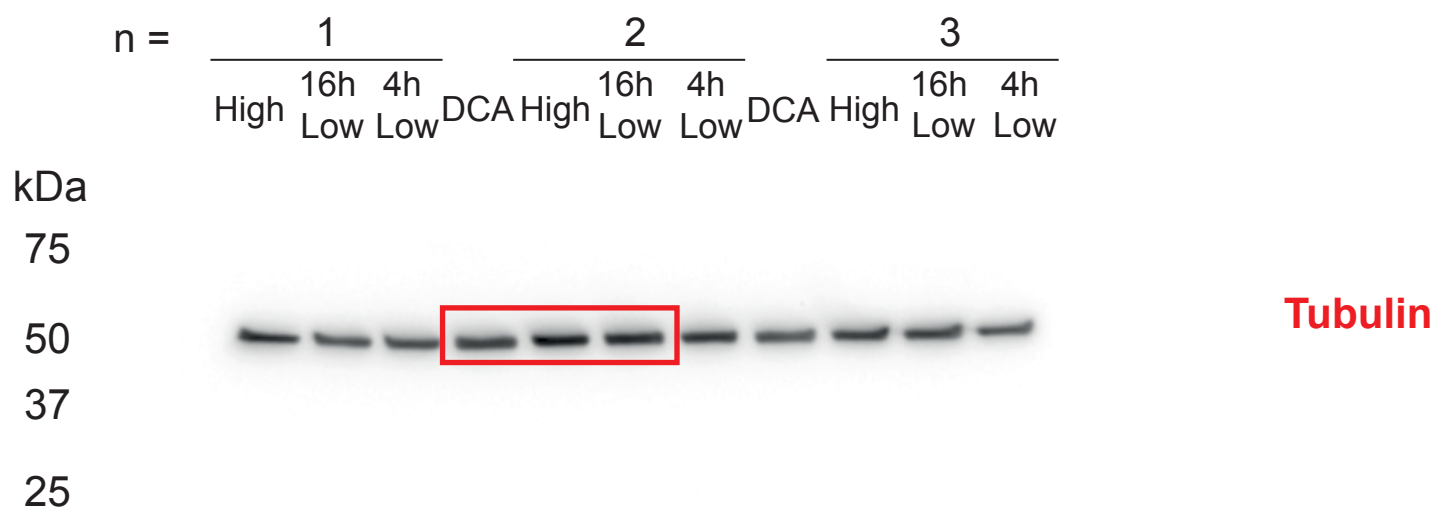

Supplement: Supplementary file 8 — Source data Fig. 2 [file 44318_2024_84_MOESM8_ESM.zip › Figure 2/2D/Western tubulin.pdf]

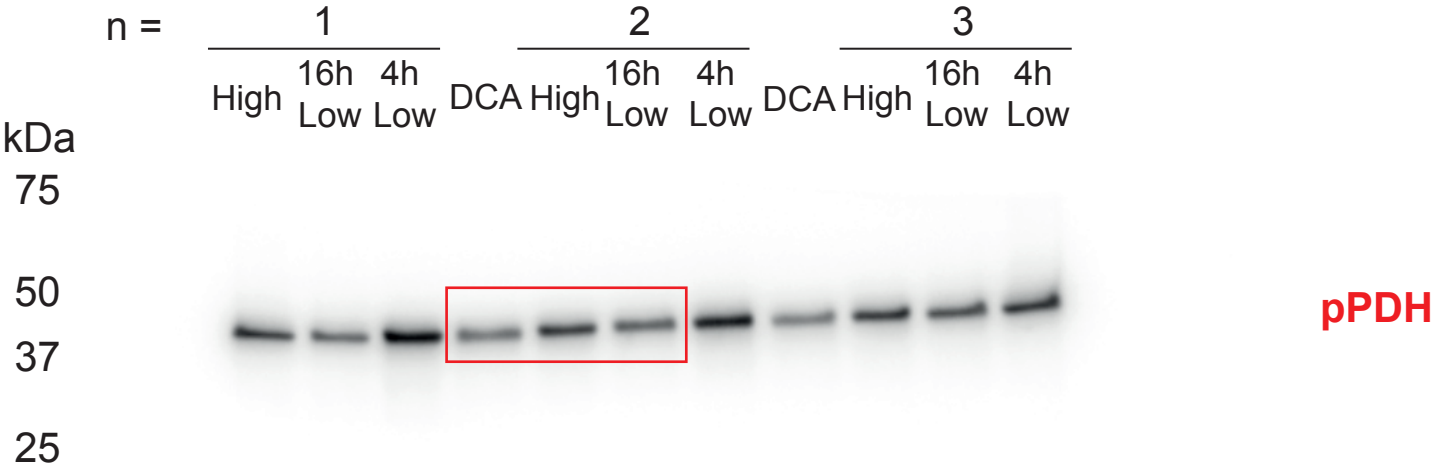

Supplement: Supplementary file 8 — Source data Fig. 2 [file 44318_2024_84_MOESM8_ESM.zip › Figure 2/2D/Western pPDH.pdf]

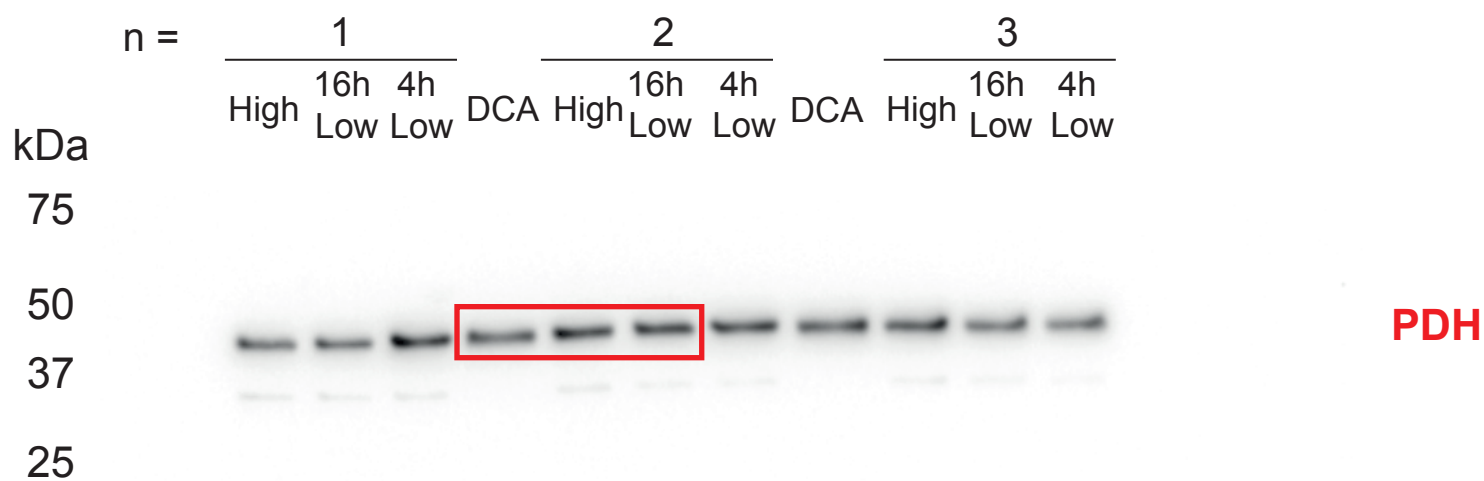

Supplement: Supplementary file 8 — Source data Fig. 2 [file 44318_2024_84_MOESM8_ESM.zip › Figure 2/2D/Western PDH.pdf]

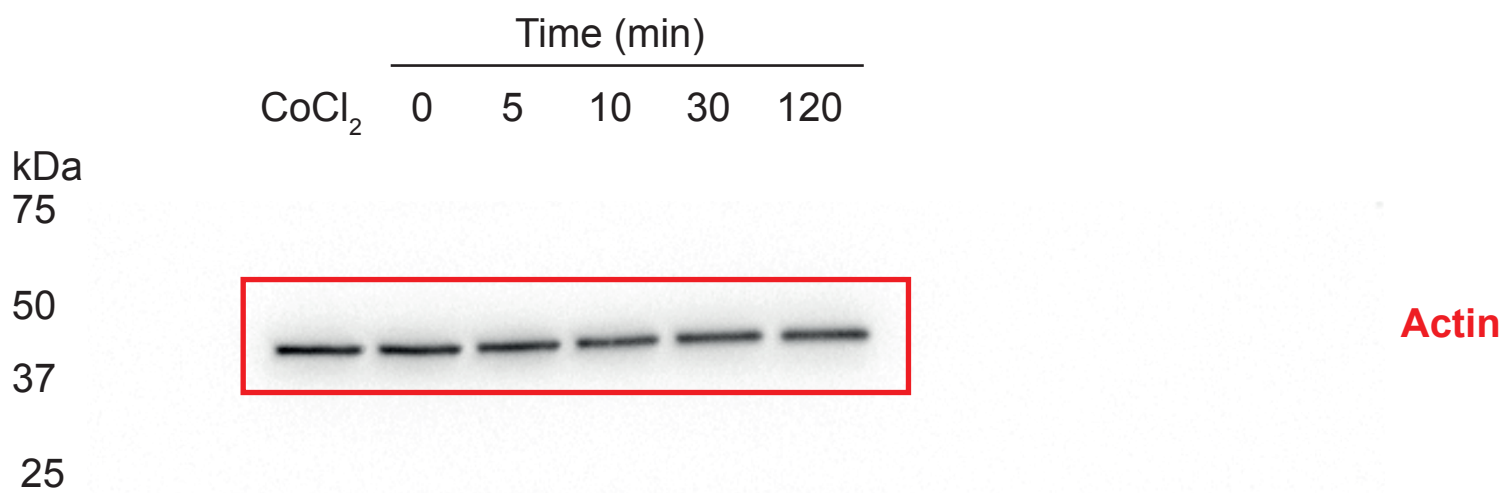

Supplement: Supplementary file 9 — Source data Fig. 3 [file 44318_2024_84_MOESM9_ESM.zip › Figure 3/3B/Western Actin.pdf]

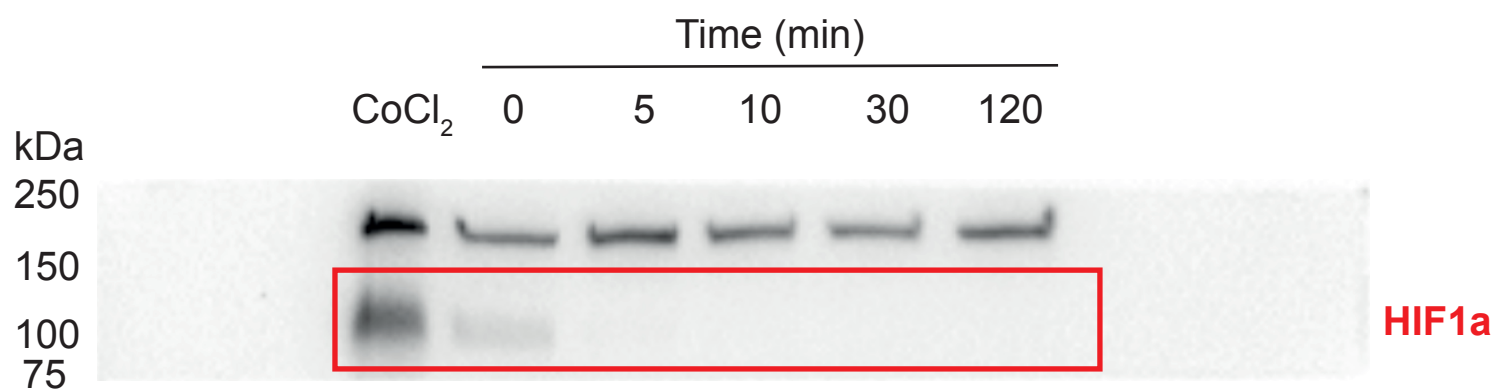

Supplement: Supplementary file 9 — Source data Fig. 3 [file 44318_2024_84_MOESM9_ESM.zip › Figure 3/3B/Western HIF1a.pdf]

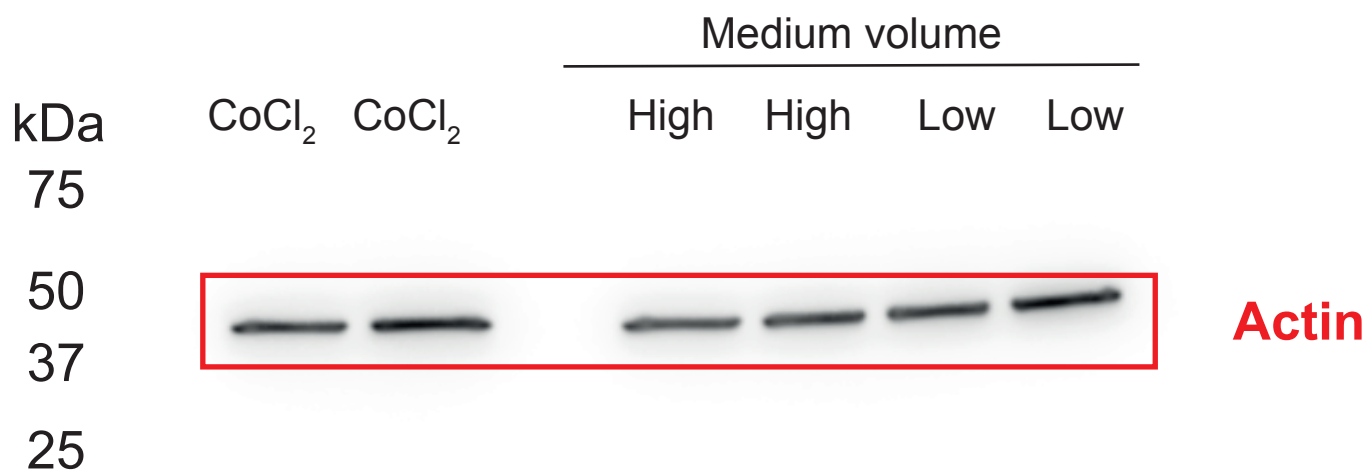

Supplement: Supplementary file 9 — Source data Fig. 3 [file 44318_2024_84_MOESM9_ESM.zip › Figure 3/3C/Western Actin.pdf]

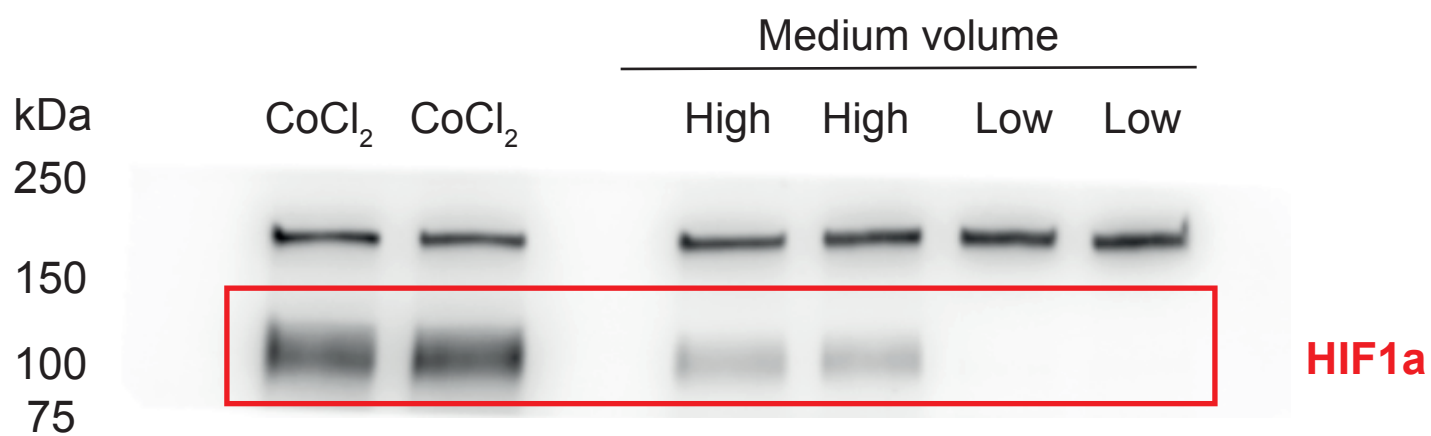

Supplement: Supplementary file 9 — Source data Fig. 3 [file 44318_2024_84_MOESM9_ESM.zip › Figure 3/3C/Western HIF1a.pdf]

High Low High Low High Low High Low

kDa  
75

50

37

25

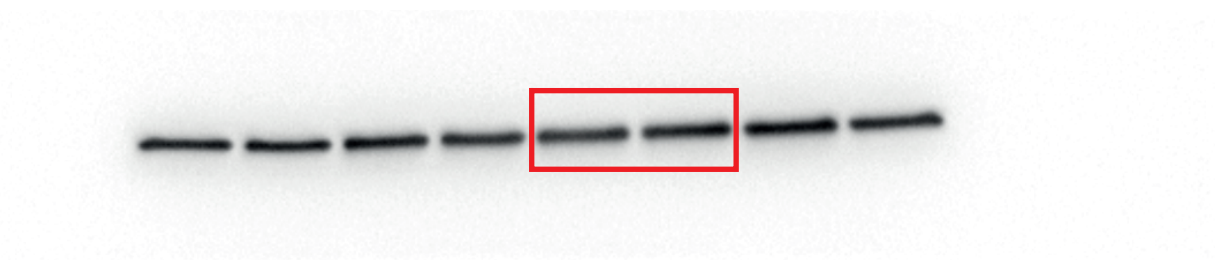

**Actin**

Supplement: Supplementary file 10 — Source data Fig. 4 [file 44318_2024_84_MOESM10_ESM.zip › Figure 4/4G/Western Actin.pdf]

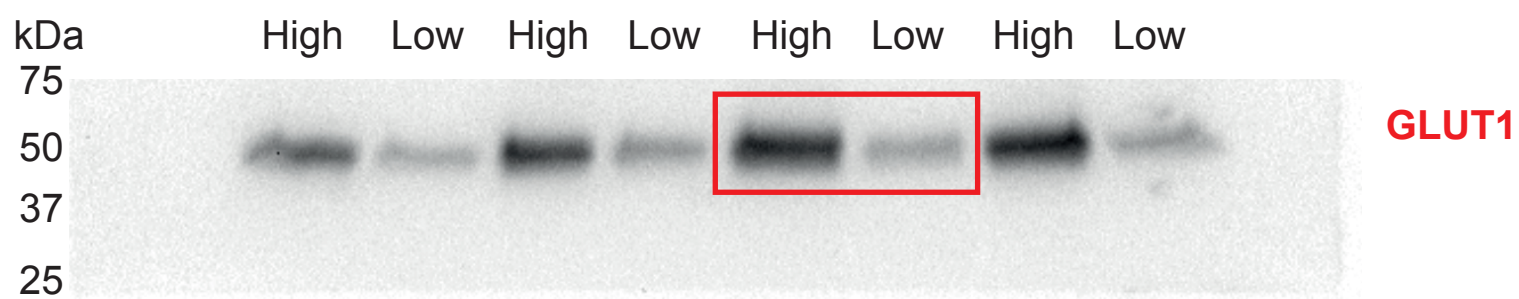

Supplement: Supplementary file 10 — Source data Fig. 4 [file 44318_2024_84_MOESM10_ESM.zip › Figure 4/4G/Western GLUT1.pdf]

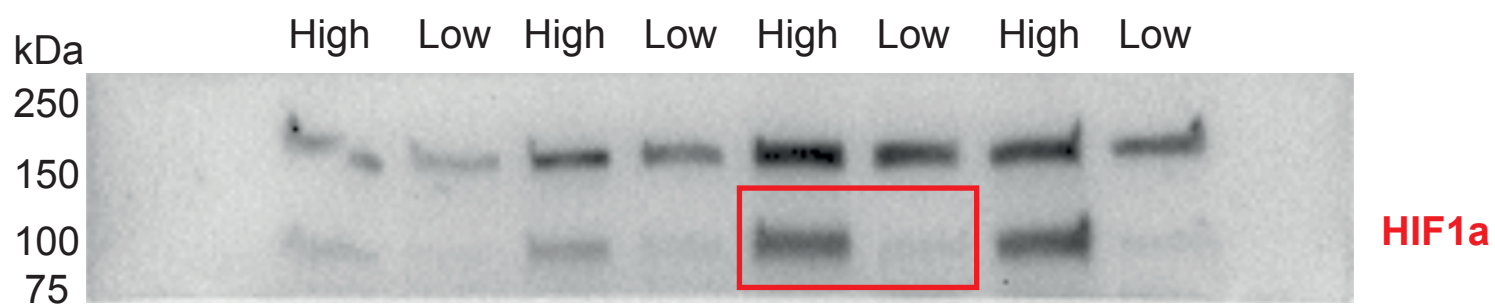

Supplement: Supplementary file 10 — Source data Fig. 4 [file 44318_2024_84_MOESM10_ESM.zip › Figure 4/4G/Western HIF1a.pdf]

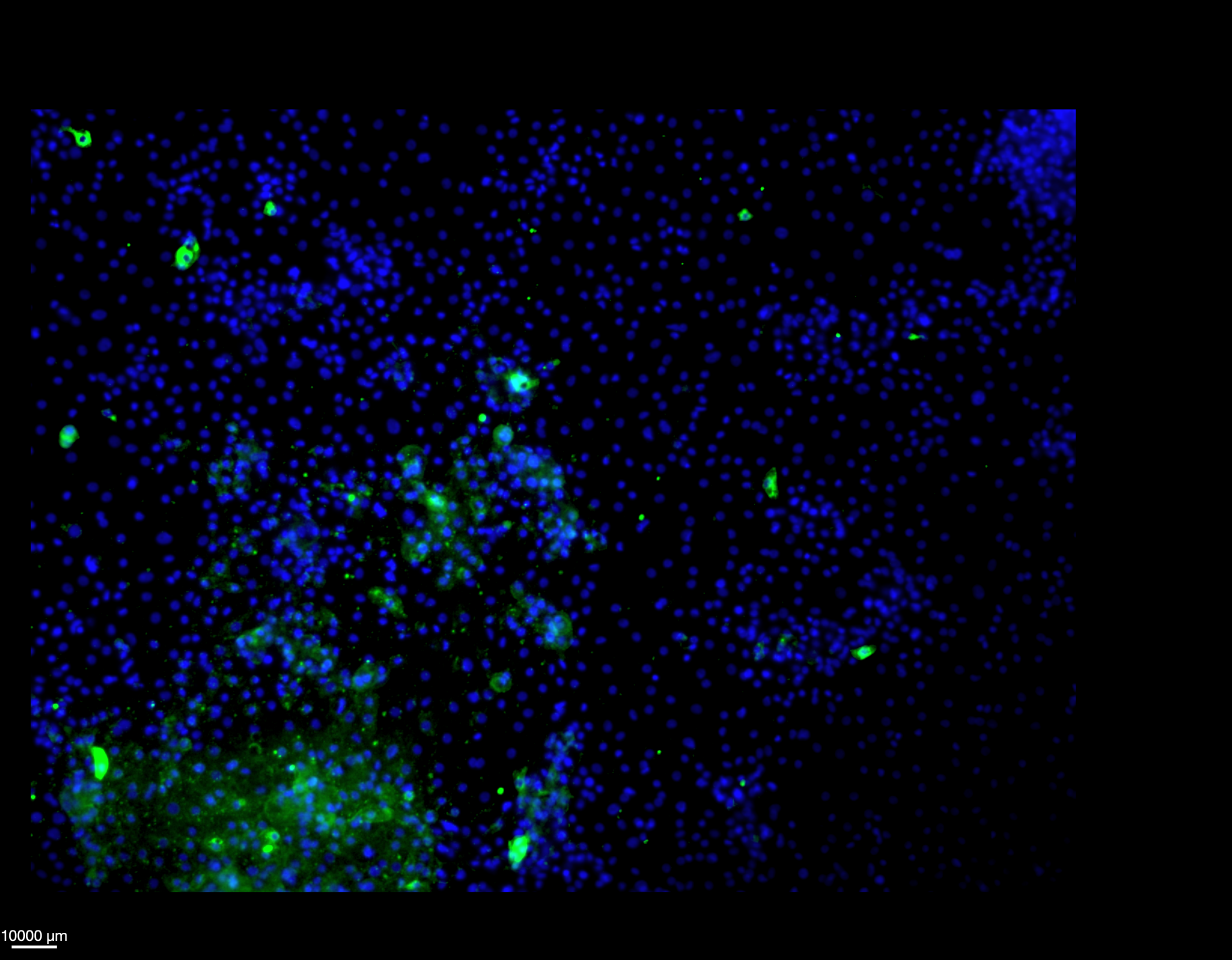

Supplement: Supplementary file 11 — Source data Fig. 5 [file 44318_2024_84_MOESM11_ESM.zip › Figure 5/5H/Albumin+DAPI High.tif]

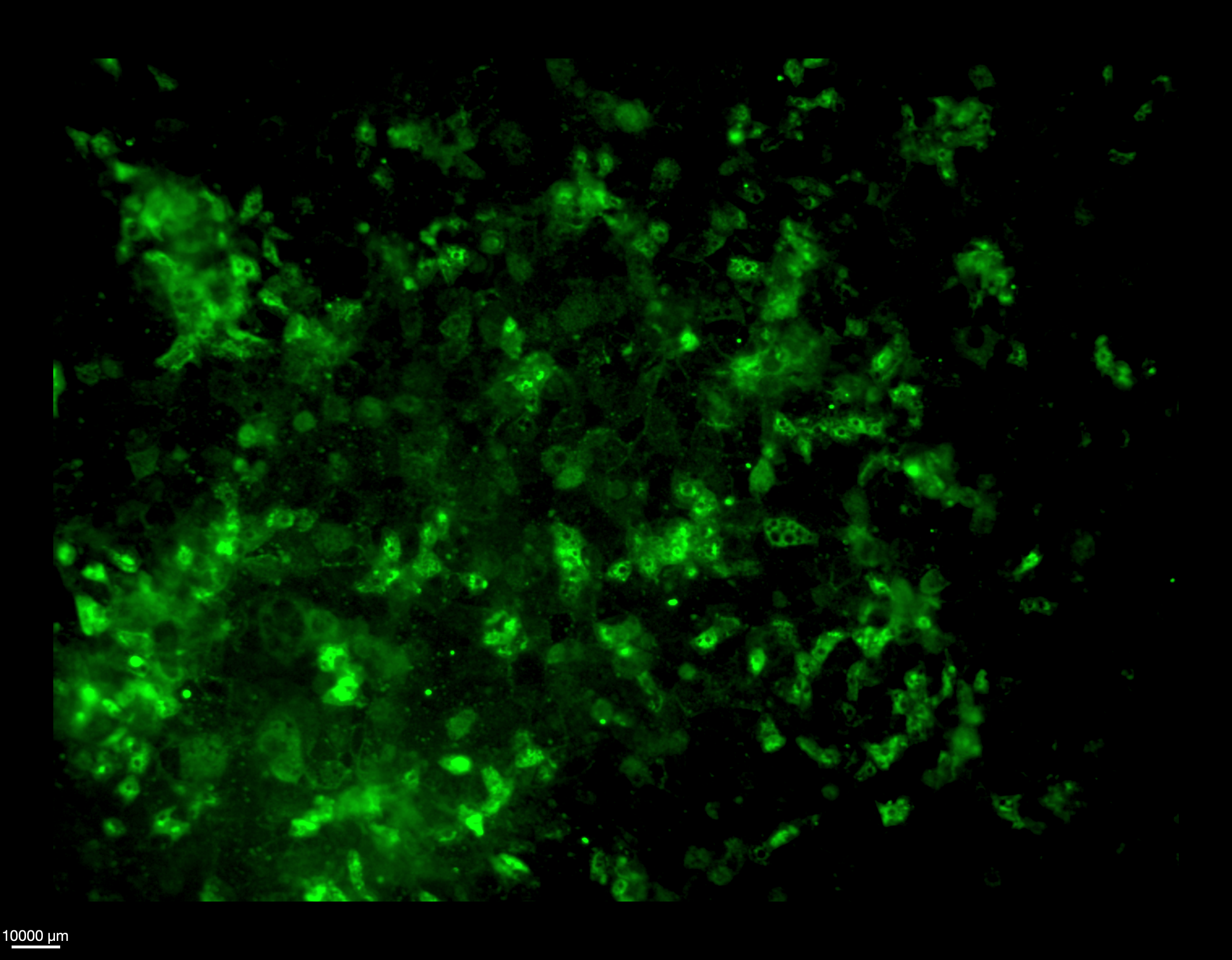

Supplement: Supplementary file 11 — Source data Fig. 5 [file 44318_2024_84_MOESM11_ESM.zip › Figure 5/5H/Albumin Low.tif]

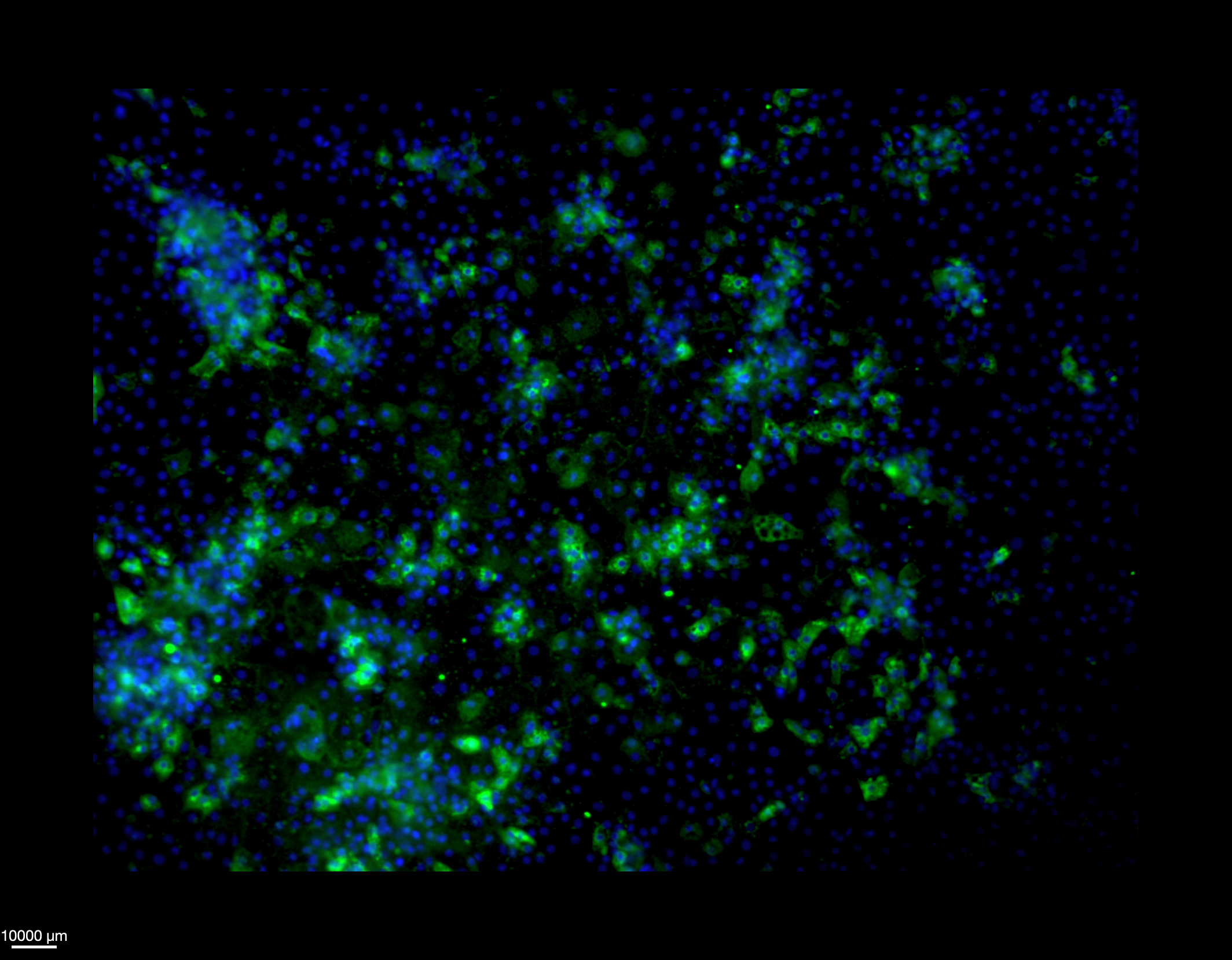

Supplement: Supplementary file 11 — Source data Fig. 5 [file 44318_2024_84_MOESM11_ESM.zip › Figure 5/5H/Albumin+DAPI low.tif]

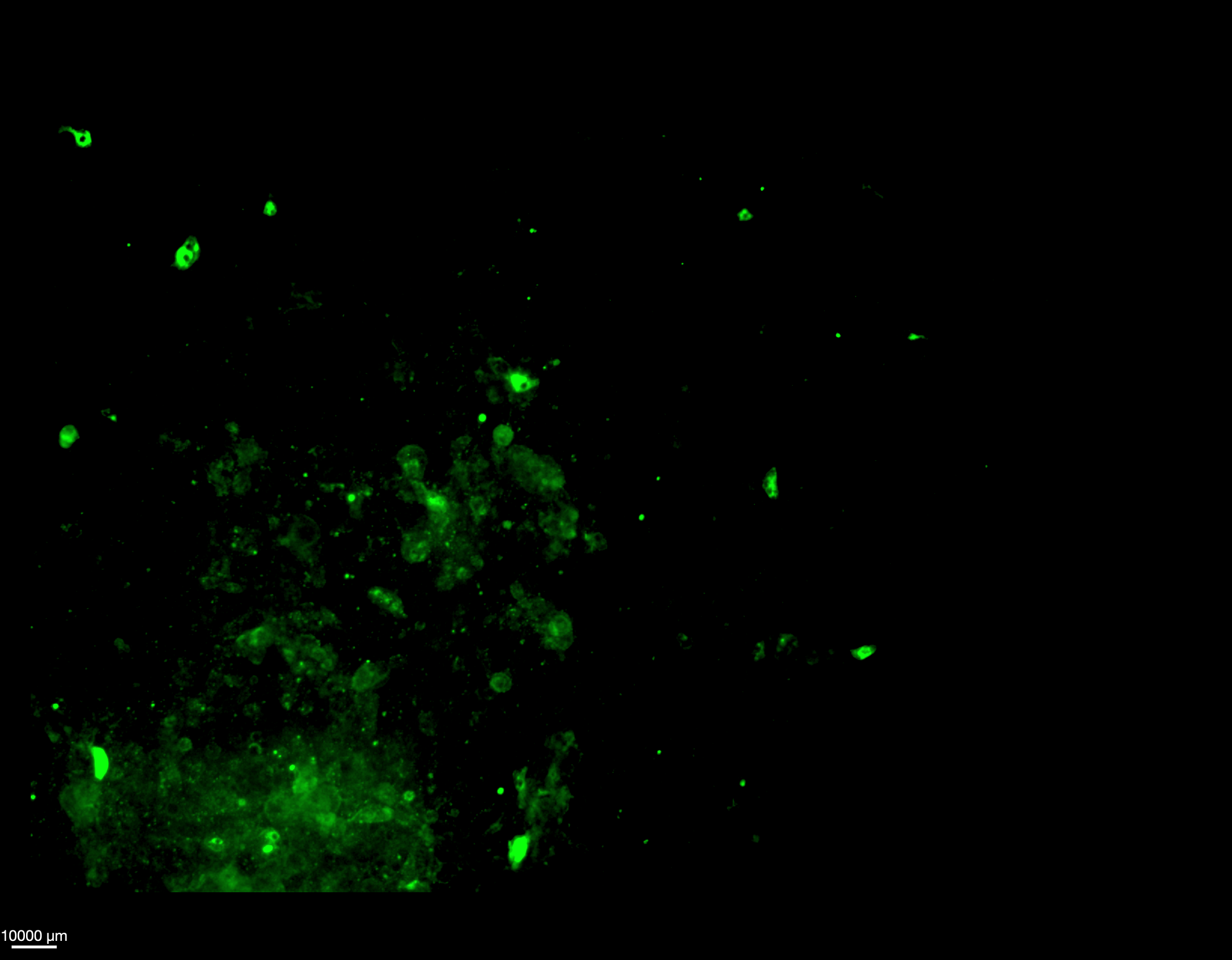

Supplement: Supplementary file 11 — Source data Fig. 5 [file 44318_2024_84_MOESM11_ESM.zip › Figure 5/5H/Albumin High.tif]
