# Supplementary material for: Limited oxygen in standard cell culture alters metabolism and function of differentiated cells
Source: EMBO J. 2024 Apr 5;43(11):4. doi: 10.1038/s44318-024-00084-7 (PMC11148168; doi:10.1038/s44318-024-00084-7)
Supplement: Supplementary file 13 — Expanded View Figures [file 44318_2024_84_MOESM13_ESM.pdf]

Expanded View Figures

Figure EV1. Changes in glucose metabolism in low medium is due to increased oxygen availability.

(**A**) Table of medium volumes used in this study and the corresponding medium heights of both top and bottom menisci. Images of each plate-type containing different medium volumes were used to measure menisci heights. Known well diameters were used to convert menisci heights from pixels to mm. 'High' refers to the standard culture volumes used. (**B**) Representative trace of fluorescence intensity indicative of pericellular oxygen concentrations under different medium volumes, measured for 24 h in 96-well plates. The bar graph shows relative oxygen levels taken at 16 h (arrow in representative trace) ($n = 4$ biological replicates). AA antimycin A. (**C**) Western blot of mitochondrial respiratory complexes I–V after 16 h of medium volume change in 12-well plates ($n = 3$ biological replicates). (**D**) Oxygen consumption rate (OCR) of permeabilised 3T3-L1 adipocytes upon different substrate stimulation after 16 h of medium volume change ($n = 5$ biological replicates). (**E**) Extracellular medium glucose and lactate measurements in 24-well or Lumox plates after 16 h culture in high or low medium volumes ($n = 3$ biological replicates). (**F**) Medium glucose concentration after 16 h of medium volume change in 12-well plates ($n = 6$ biological replicates). (**G**) Extracellular medium glucose and lactate measurements after 16 h medium volume change with different starting glucose concentrations in 12-well plates. ($n = 4$ biological replicates). (**H**) OCR measurements from different cell densities in 96-well plates ($n = 3$ biological replicates). (**I**) Extracellular medium glucose and lactate measurements after 16 h medium volume change with different cell densities in 12-well plates measured by DNA concentration. ($n = 3$ biological replicates). Data information: Data were represented as mean ± SEM (**B**, **D–I**). ****$p < 0.0001$ by two-way ANOVA with Šidák correction for multiple comparisons (**E**).

© The Author(s)

A

Plate	12-well			24-well			96-well		
Well area (cm²)	3.8			1.9			0.32		
	Medium volume (μL)	Top men. (mm)	Bottom men. (mm)	Medium volume (μL)	Top men. (mm)	Bottom men. (mm)	Medium volume (μL)	Top men. (mm)	Bottom men. (mm)
High (standard)	1000	4.7	2.4	500	3.9	2.2	100	3.3	2.5
Mid	666	3.8	1.7	333	3.5	1.6	50	2.2	1.5
Low	333	3.0	0.8	167	1.9	0.8	33	2.0	0.7

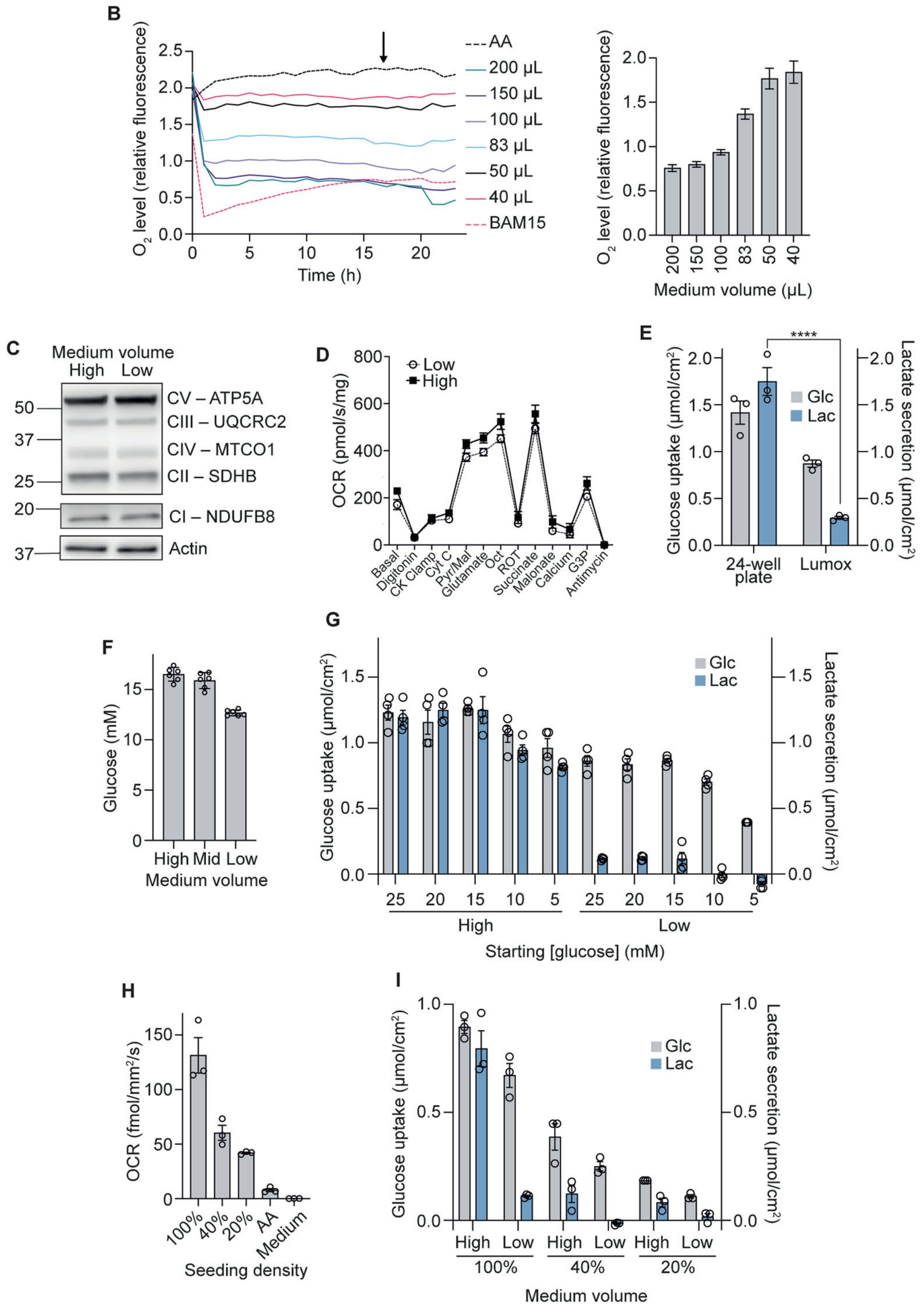

 © The Author(s)

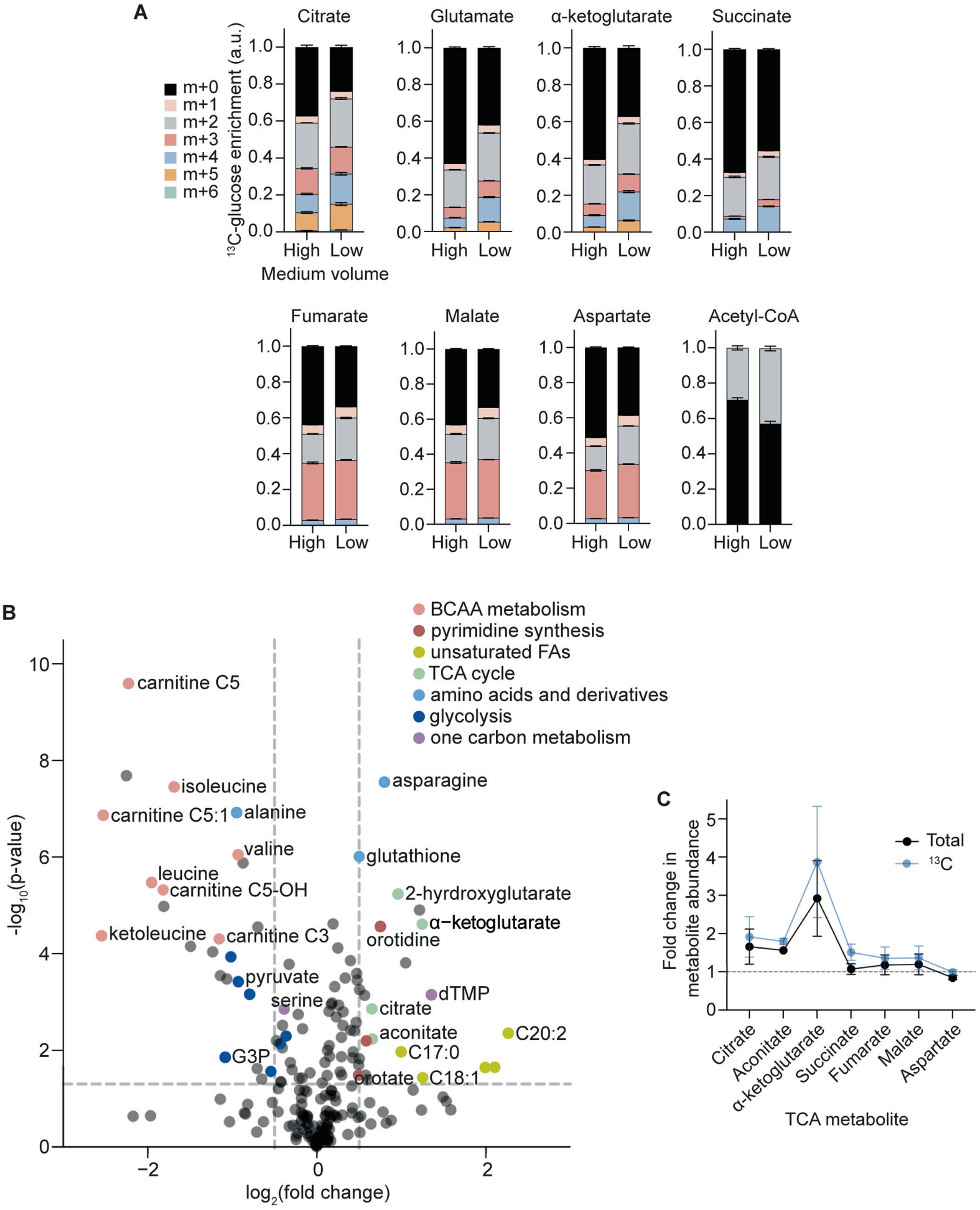
A
Citrate
Glutamate
α-ketoglutarate
Succinate
m+0
m+1
m+2
m+3
m+4
m+5
m+6
13C-glucose enrichment (a.u.)
High Low
Medium volume
Fumarate
Malate
Aspartate
Acetyl-CoA
B
BCAA metabolism
pyrimidine synthesis
unsaturated FAs
TCA cycle
amino acids and derivatives
glycolysis
one carbon metabolism
carnitine C5
isoleucine
carnitine C5:1
alanine
asparagine
valine
glutathione
leucine
carnitine C5-OH
2-hyrdroxyglutarate
α−ketoglutarate
ketoleucine
carnitine C3
orotidine
pyruvate
serine
dTMP
citrate
aconitate
C20:2
G3P
C17:0
orotate
C18:1
-log10(p-value)
log2(fold change)
C
Total
13C
Fold change in metabolite abundance
Citrate
Aconitate
α-ketoglutarate
Succinate
Fumarate
Malate
Aspartate
TCA metabolite

◀ **Figure EV2. Lowering medium volumes rewires cellular metabolism.**

(**A**) Fractional abundance of each isotopologue after 4 h medium volume change ($n = 6$ biological replicates). (**B**) Volcano plot of differentially regulated metabolites after 16 h medium volume change. Metabolites of interest which are significantly changed ($p < 0.05$) are highlighted according to their metabolic pathways ($n = 6$ biological replicates). BCAA branched-chain amino acid, FA fatty acid, G3P glyceraldehyde−3−phosphate. (**C**) Fold change of total and U^{13}C-glucose labelled TCA metabolite abundance after 16 h medium volume change in 12-well plates ($n = 4$ biological replicates). Data information: Data were represented as mean ± SEM (**A**, **C**). p value threshold of 0.05 (**B**) was determined using differential metabolite analysis (DMA) with Student's t-test.

 © The Author(s)

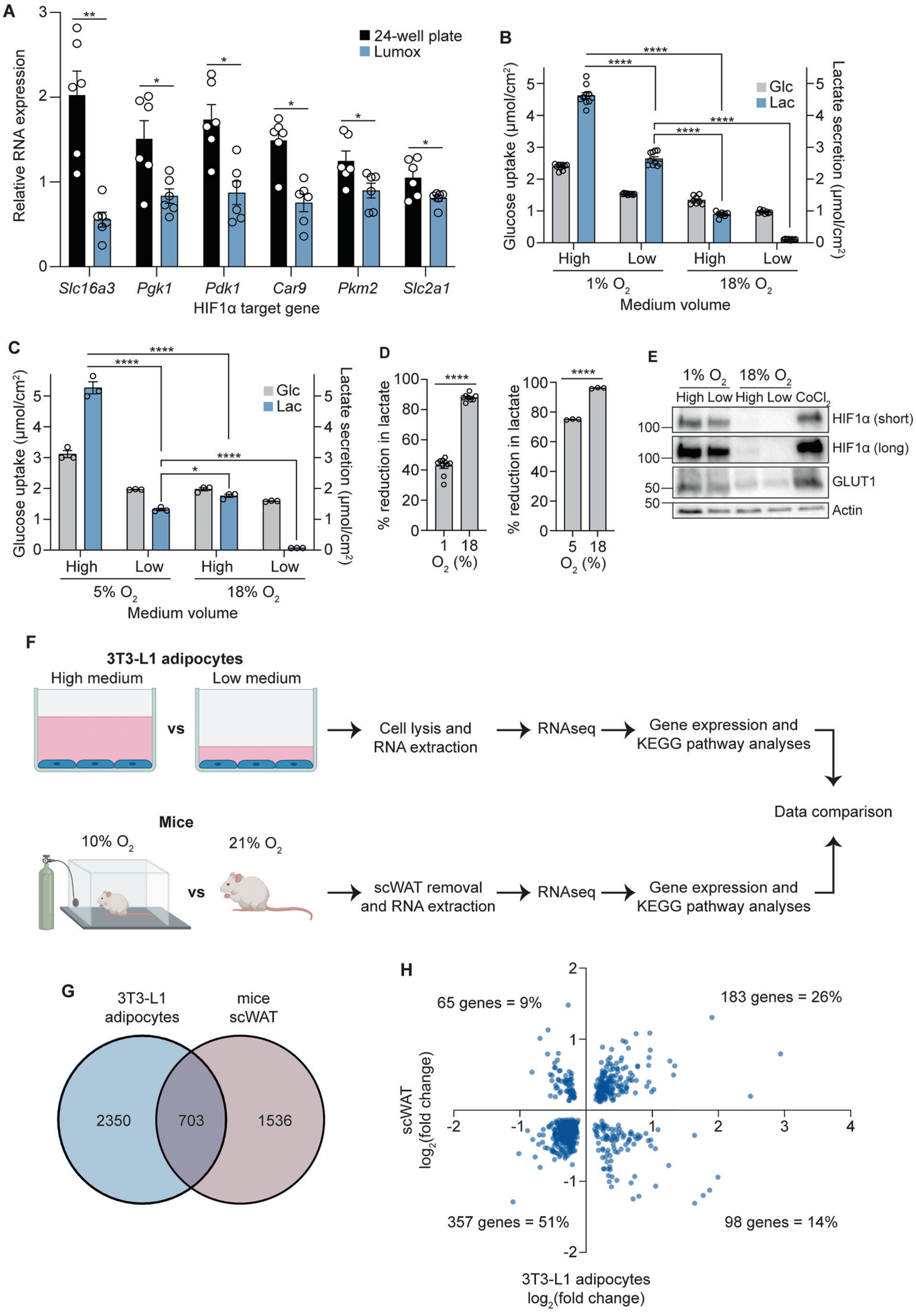
A
24-well plate
Lumox
Relative RNA expression
Slc16a3
Pgk1
Pdk1
Car9
Pkm2
Slc2a1
HIF1α target gene
B
Glc
Lac
Glucose uptake (µmol/cm²)
Lactate secretion (µmol/cm²)
High
Low
1% O2
18% O2
Medium volume
C
5% O2
18% O2
D
% reduction in lactate
O2 (%)
E
1% O2
18% O2
High Low High Low CoCl2
HIF1α (short)
HIF1α (long)
GLUT1
Actin
F
3T3-L1 adipocytes
High medium
Low medium
vs
Cell lysis and RNA extraction
RNAseq
Gene expression and KEGG pathway analyses
Data comparison
Mice
10% O2
21% O2
scWAT removal and RNA extraction
G
3T3-L1 adipocytes
mice scWAT
2350
703
1536
H
65 genes = 9%
183 genes = 26%
357 genes = 51%
98 genes = 14%
scWAT log2(fold change)
3T3-L1 adipocytes log2(fold change)

Figure EV3. Reducing oxygen availability causes metabolic and transcriptional rewiring.

(**A**) Relative RNA expression of HIF1α target genes in 3T3-L1 adipocytes cultured in either 24-well or gas-permeable Lumox plates ($n = 6$ biological replicates). (**B**) Extracellular medium glucose and lactate measurements after 16 h medium volume change (12-well plate) in 1 or 18% oxygen incubators ($n = 8$–10 biological replicates). (**C**) Extracellular medium glucose and lactate measurements after 16 h medium volume change (12-well plate) in 5 or 18% oxygen incubators ($n = 3$ biological replicates). (**D**) Percentage reduction in lactate production calculated from Fig. EV3B (1% O_2) ($n = 8$–10 biological replicates) and EV3C (5% O_2) ($n = 3$ biological replicates). (**E**) Western blot of HIF1α (after both short and long imaging exposures) and GLUT1 after 16 h medium volume change at 1 or 18% O_2 in 12-well plates ($n = 3$ biological replicates). (**F**) Schematic representation of the RNAseq experimental workflow. RNA extracted from 3T3-L1 adipocytes (cultured in high or low medium for 16 h in 12-well plates) or scWAT (obtained from mice kept in 10 or 21% O_2 for 4 weeks) were sequenced. The two sets of analysed data were then compared. (**G**) Venn diagram showing the overlapping differentially expressed genes (p-adj < 0.05) from both 3T3-L1 adipocytes (high *vs* low medium) ($n = 6$ biological replicates) and mice scWAT (10 vs 21% O_2) ($n = 10$ biological replicates). (**H**) Fold change of the 703 differentially expressed genes in 3T3-L1 adipocytes (y-axis) and mice scWAT (x-axis) from the intersection in Fig. EV3D, showing a 77% directional concordance. Data information: Data were represented as mean ± SEM (**A–D**). *$p < 0.05$, **$p < 0.01$, ****$p < 0.0001$ by two-way ANOVA with Šidák correction for multiple comparisons (**B**, **C**), or by paired/unpaired Student's *t*-tests (**A**, **D**).

 © The Author(s)

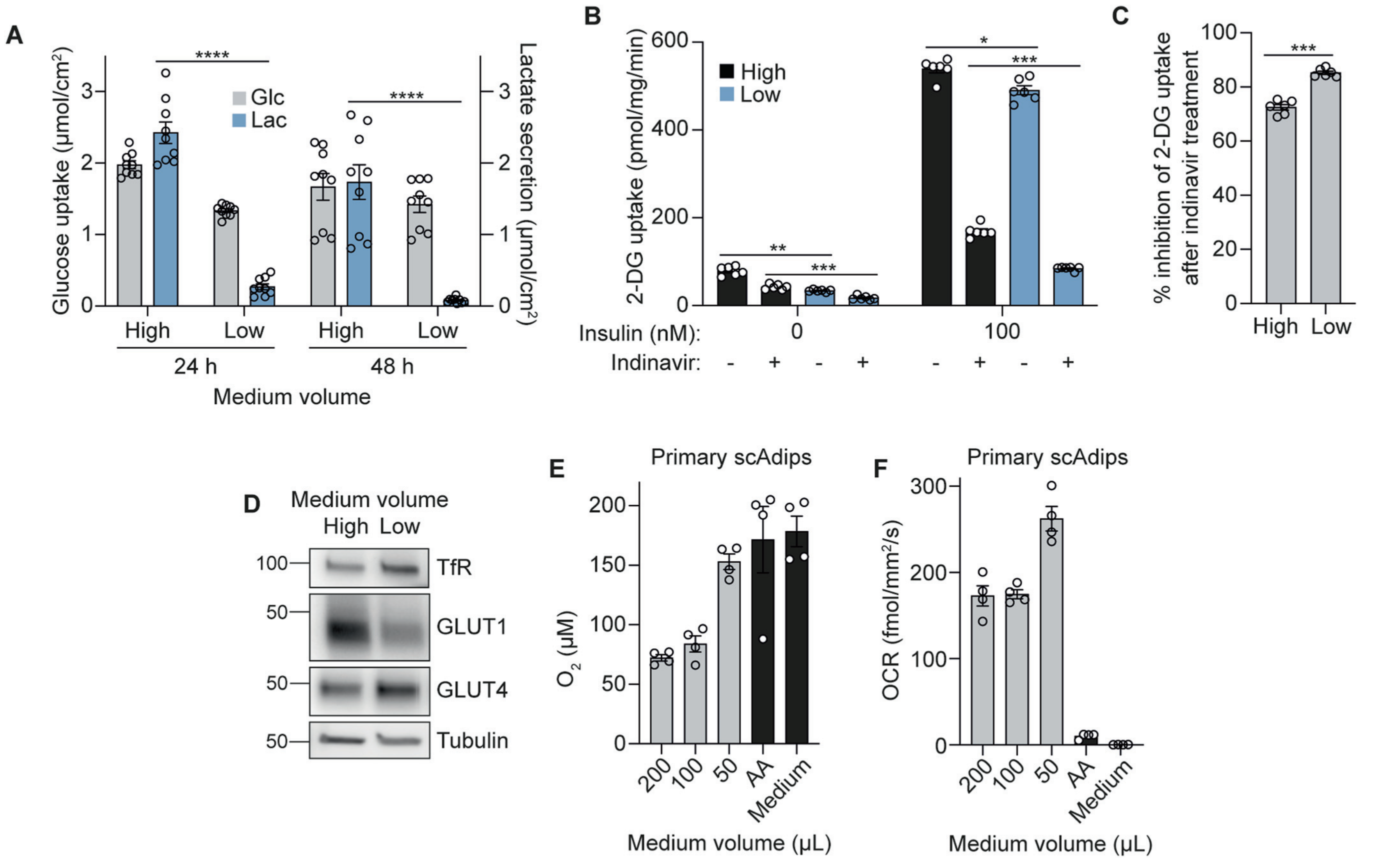

Figure EV4. Effects of lowering medium volumes on 3T3-L1 adipocyte glucose metabolism and oxygen use in primary scAdips.

(**A**) Extracellular measurements of 3T3-L1 medium glucose and lactate 24 or 48 h after medium volume change in 12-well plates ($n = 3$ technical replicates from $n = 3$ biological replicates). (**B**) 2-deoxyglucose (DG) uptake after insulin stimulation and 200 µM indinavir (GLUT4 inhibitor) treatment. Cells were cultured in high or low medium for 48 h in 24-well plates prior to the experiment ($n = 6$ biological replicates). (**C**) Percentage inhibition of 2-DG uptake after indinavir treatment, calculated from the difference between +/− indinavir treated conditions, as a percentage of -indinavir 2-DG uptake upon 100 nM insulin stimulation. Graph shows the percentage of 2-DG uptake that is GLUT4-dependent (i.e. inhibited by indinavir) ($n = 6$ biological replicates). (**D**) Western blot of GLUT1 and GLUT4 in 3T3-L1s after 48 h medium volume change (12-well plate) ($n = 6$ biological replicates). (**E**) The pericellular oxygen concentration of primary scAdips cultured with different medium volumes in 96-well plates ($n = 4$ biological replicates). AA antimycin A. (**F**) OCR of primary scAdips cultured with different medium volumes in 96-well plates ($n = 4$ biological replicates). Data information: Data were represented as mean ± SEM (**A–D**). *$p < 0.05$, **$p < 0.01$, ***$p < 0.001$, ****$p < 0.0001$ by two-way ANOVA with Šidák correction for multiple comparisons (**A**. **B**), or by paired two-tailed Student's *t*-test (**C**).

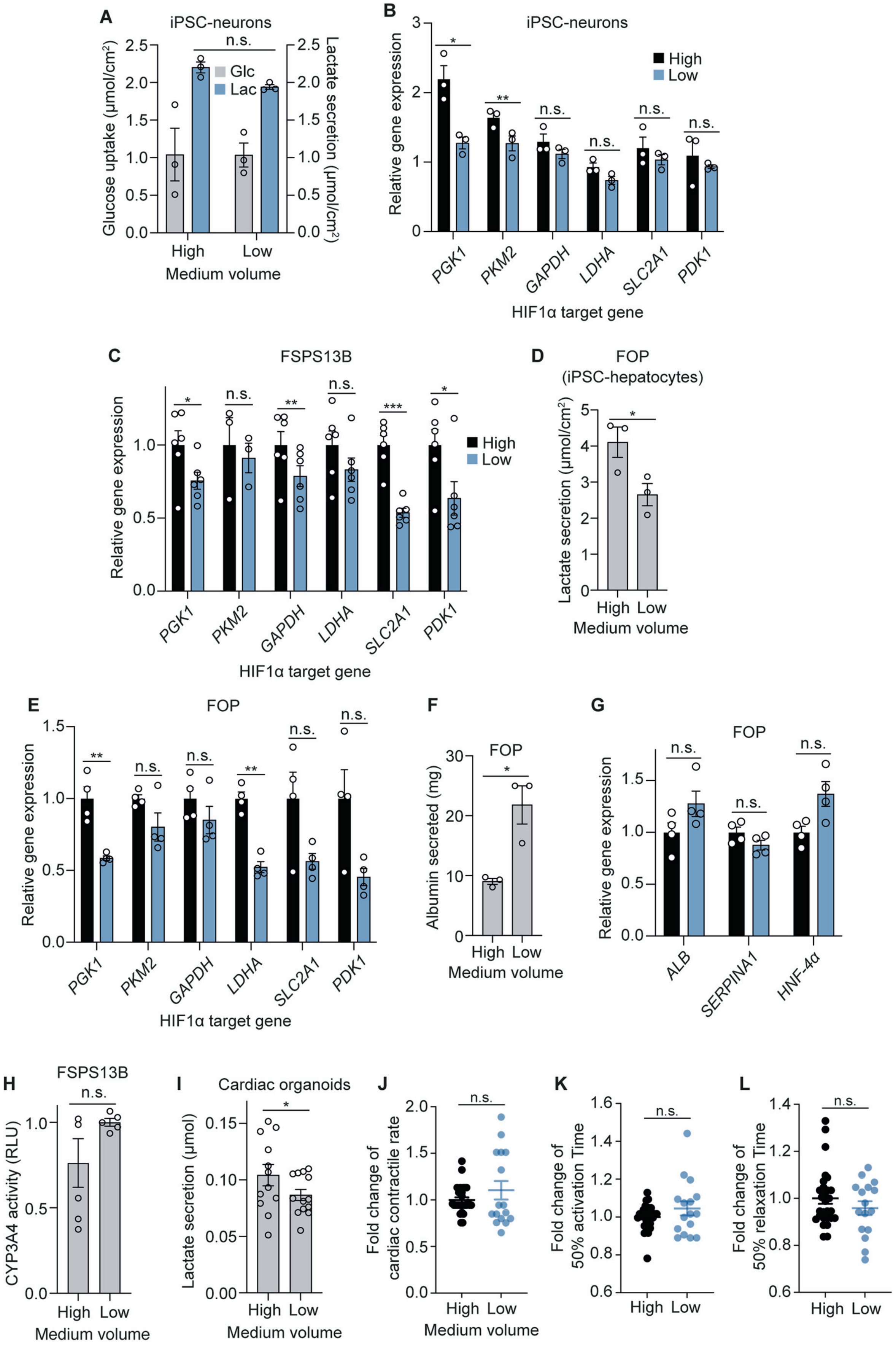
A
iPSC-neurons
n.s.
Glc
Lac
Glucose uptake (μmol/cm²)
Lactate secretion (μmol/cm²)
High
Low
Medium volume
B
iPSC-neurons
High
Low
Relative gene expression
PGK1
PKM2
GAPDH
LDHA
SLC2A1
PDK1
HIF1α target gene
C
FSPS13B
Relative gene expression
D
FOP
(iPSC-hepatocytes)
Lactate secretion (μmol/cm²)
E
FOP
F
FOP
Albumin secreted (mg)
G
FOP
ALB
SERPINA1
HNF-4α
H
FSPS13B
CYP3A4 activity (RLU)
I
Cardiac organoids
Lactate secretion (μmol)
J
Fold change of cardiac contractile rate
K
Fold change of 50% activation Time
L
Fold change of 50% relaxation Time

 © The Author(s)

Figure EV5. Effects of low medium volumes on other primary and hPSC-derived cell types.

(**A**) Extracellular medium glucose and lactate measurements from iPSC-derived neurones after 16 h medium volume change in 6-well plates ($n = 3$ biological replicates). (**B**) Relative RNA expression of HIF1α target genes in iPSC-derived neurones after 16 h medium volume change in 12-well plates ($n = 3$ biological replicates). (**C**) Relative RNA expression of HIF1α target genes in FSPS13B hepatocytes cultured under different medium volumes in 12-well plates throughout differentiation ($n = 3$–6 biological replicates). (**D**) Lactate secretion in FOP hepatocytes after medium volume change in 12-well plates throughout differentiation ($n = 3$ biological replicates). (**E**) Relative RNA expression of HIF1α target genes in FOP hepatocytes cultured under different medium volumes in 12-well plates throughout differentiation ($n = 4$ biological replicates). (**F**) Albumin secretion over 24 h by FOP hepatocytes after medium volume change in 12-well plates throughout differentiation ($n = 3$ biological replicates). (**G**) Relative RNA expression of hepatocyte differentiation marker genes in FOP hepatocytes after medium volume change in 12-well plates throughout differentiation ($n = 4$ biological replicates). (**H**) Relative CYP3A4 activity in FSPS13B hepatocytes after medium volume change throughout differentiation ($n = 5$ biological replicates). (**I**) Lactate secretion by cardiac organoids after 48 h of medium volume change ($n = 3$–6 technical replicates from $n = 3$ biological replicates). High = 150 μL, Low = 50 μL. (**J**) Contractile rate (normalised) ($n = 2$–17 technical replicates from $n = 4$ biological replicates). (**K**) Time from 50% activation to peak (normalised) ($n = 2$–17 technical replicates from $n = 4$ biological replicates). (**L**) Time from peak to 50% relaxation (normalised) ($n = 2$–17 technical replicates from $n = 4$ biological replicates). Data information: All data were represented as mean ± SEM (**A**–**L**). n.s. non-significant; $^{*}p < 0.05$, $^{**}p < 0.01$, $^{***}p < 0.001$ by two-way ANOVA (**A**) or by paired/unpaired two-tailed Student's t-test (**B**–**L**).

© The Author(s)
